# Clinical picture, management and risk stratification in patients with cardiogenic shock: does gender matter?

**DOI:** 10.1186/s12872-020-01467-4

**Published:** 2020-04-21

**Authors:** Elena Collado-Lledó, Isaac Llaó, Mercedes Rivas-Lasarte, Victor González-Fernández, Francisco J. Noriega, Francisco José Hernández-Perez, Oriol Alegre, Alessandro Sionis, Rosa M. Lidón, Ana Viana-Tejedor, Javier Segovia-Cubero, Albert Ariza-Solé

**Affiliations:** 1 Intensive Cardiac Care Unit. Cardiology Department, Hospital Universitari de Bellvitge –IDIBELL, Feixa Llarga s/n. 08907. L’Hospitalet de Llobregat, Barcelona, Spain; 2 Intensive Cardiac Care Unit, Cardiology Department, Hospital de la Santa Creu i Sant Pau, IIB-SantPau, CIBER-CV, Universidad Autònoma de Barcelona, Barcelona, Spain; 3 Cardiovascular Critical Care Unit, Cardiology Department, Vall d’Hebron Hospital, CIBER-CV Vall d’Hebron Hospital, Barcelona, Spain; 4 Acute Cardiac Care Unit, Cardiology Department, Cardiovascular Institute, Hospital Clínico San Carlos, Madrid, Spain; 5 Advanced Heart Failure and Transplant Unit, Cardiology Department, Hospital Universitario Puerta de Hierro, CIBER-CV, Universidad Autónoma de Madrid, Madrid, Spain

**Keywords:** Cardiogenic shock, Gender, Risk stratification, Prognosis

## Abstract

**Background:**

Early recognition and risk stratification are crucial in cardiogenic shock (CS). A lower adherence to recommendations has been described in women with cardiovascular diseases. Little information exists about disparities in clinical picture, management and performance of risk stratification tools according to gender in patients with CS.

**Methods:**

Data from the multicenter Red-Shock registry were used. All consecutive patients with CS were included. Both CardShock and IABP-SHOCK II risk scores were calculated. The primary end-point was in-hospital mortality. The discriminative ability of both scores according to gender was assessed by binary logistic regression, calculating *Receiver operating characteristic* (ROC) curves and the corresponding area under the curve (AUC).

**Results:**

A total of 793 patients were included, of whom 222 (28%) were female. Women were significantly older and had a lower proportion of chronic obstructive pulmonary disease and prior myocardial infarction. CS was less often related to acute coronary syndromes (ACS) in women. The use of vasoactive drugs, renal replacement therapy, invasive ventilation, therapeutic hypothermia and mechanical circulatory support was similar between both groups. In-hospital mortality was 346/793 (43.6%). Mortality was not significantly different according to gender (*p* = 0.194).

Cardshock risk score showed a good ability for predicting in-hospital mortality both in man (AUC 0.69) and women (AUC 0.735). Likewise, the IABP-II successfully predicted in-hospital mortality in both groups (man: AUC 0.693; women: AUC 0.722).

**Conclusions:**

No significant differences were observed regarding management and in-hospital mortality according to gender. Both the CardShock and IABP-II risk scores depicted a good ability for predicting mortality also in women with CS.

## Background

Cardiogenic shock (CS) is a severe clinical condition that leads to a high mortality (40–59%) despite the treatment advances made in the last decade, including early myocardial reperfusion in myocardial infarction (MI) [1–3]. Early recognition and risk stratification are crucial for both patient allocation and selection of the optimal treatment strategy [4]. The recently developed CardShock [1] and IABP-SHOCK II [5] risk scores contain variables that can be easily obtained at the bedside or with routine laboratory tests, and have been recently validated in a large cohort of non-selected patients with CS from a Spanish multicenter registry.

On the other hand, significant differences regarding clinical profile, management and prognosis according to gender have been previously described in patients with cardiovascular disease [6–9]. Importantly a significantly lower adherence to current recommendations has been consistently described in other clinical scenarios, especially in patients with Acute Coronary Syndromes (ACS) [10, 11]. Little information exists about clinical picture, management and prognosis according to gender in patients with CS.

Therefore, the aim of this study was to describe the clinical picture, management and the predictive ability of the Cardshock and IABP-II risk scores according to gender in patients with CS from routine clinical practice.

## Methods

### Study design and inclusion criteria

Red-Shock registry [12] is an observational, retrospective, cohort study. The design has been previously described [12]. Consecutive patients aged 18 years or older with cardiogenic shock ((a) systolic blood pressure < 90 mmHg (after adequate fluid challenge) for 30 min or need for vasopressor therapy to maintain systolic blood pressure > 90 mmHg, and b) signs of hypoperfusion) were included. Patients with shock after cardiac or non-cardiac surgery or ongoing haemodynamically significant arrhythmia as the cause of hypotension were excluded.

The study was approved by the reference research ethics committee and was conducted in accordance with the Declaration of Helsinki. Because the study was retrospective, informed consent was not required. Patients were treated according to the current practice of each center.

### Data collection

Demographic characteristics, clinical profile at admission (within 6 h after the onset of CS) and treatments administered were recorded.

Both the CardShock [1] and the IABP-SHOCK II [5] risk scores were calculated in the study cohort. An optimal thrombolysis in myocardial infarction (TIMI) flow was considered by default (0 points) in non ACS patients.

### Outcomes

Main outcome measured was in-hospital mortality according to gender status.

### Statistical analysis

Baseline characteristics and clinical management were assessed according to gender. Categorical variables were reported as frequencies and percentages, and statistical differences were analysed by using the χ2 test. Continuous variables were reported as the mean and standard deviation or the median and interquartile range; statistical differences were analysed using the Student t test or Wilcoxon test, as appropriate.

The discriminative ability of the CardShock score and the IABP-II score were assessed by a binary regression logistic model, calculating Receiver operating characteristic (ROC) curves and the corresponding area under the curve (AUC). For the performance of this analysis only patients with available Cardshock and IABP-II scores values were included (*n* = 696). Comparison between AUCs was performed by the DeLong method [13]. Calibration of the scores was assessed by the Hosmer-Lemeshow test. A *P*-value < 0.05 was considered significant and all statistical analyses were performed using STATA software, version 13.1 (Stata Corp, College Station, TX, USA).

#### Ethics statement

All procedures performed in this study were in accordance with the ethical standards of the institutional research committee and with the 1964 Helsinki declaration and its later amendments or comparable ethical standards. This study was approved by the reference institutional ethics comittee).

## Results

A total of 793 patients with CS were included from 6 centers, of whom 222 (28%) were female. Mean age was 65.1 ± 15 years. A high proportion of comorbidities was observed, such as hypertension (58.8%) diabetes mellitus (39.6%), prior MI (20.1%) or previous heart failure (26.9%). In most cases (501/793, 63.2%) CS was related to ACS.

Patients had a high risk profile, with a severely depressed left ventricular function, high proportion of confusion at presentation, elevated lactate values and low values of estimated glomerular filtration (eGFR) (Table 1).

**Table 1:** Clinical characteristics according to gender status

	Whole cohort (*n* = 793)	Male (*n* = 571)	Female (*n* = 222)	*P* value
Age	65.1 (15)	64.2 (14)	67.3 (16)	0,013
BMI	26.9 (5)	27 (4)	26.7 (6)	0.537
Diabetes mellitus	314 (39.6)	226 (39.6)	88 (39.6)	0.988
Hypertension	466 (58.8)	331 (58)	135 (61.1)	0.424
Dyslipidemia	446 (56.2)	329 (57.6)	117 (52.7)	0.210
Active smoker	219 (27.7)	190 (33.4)	29 (13.1)	0.001
COPD	130 (16.4)	108 (18.9)	22 (9.9)	0.002
Prior stroke	69 (8.7)	54 (9.5)	15 (6.8)	0.226
PAD	90 (11.3)	71 (12.4)	19 (8.6)	0.122
Prior MI	159 (20.1)	126 (22.1)	33 (14.9)	0.023
Prior PCI	96 (12.1)	74 (13)	22 (9.9)	0.485
Prior CABG	33 (4.2)	24 (4.2)	9 (4.1)	0.789
Renal failure	134 (16.9)	104 (18.2)	30 (13.5)	0.113
Previous heart failure	213 (26.9)	150 (26.3)	63 (28.5)	0.524
ACS-related cardiogenic shock	501 (63.2)	373 (65.3)	128 (57.7)	0.044
Systolic blood pressure	81 (16)	81 (16)	80 (15)	0.969
Heart rate	94 (28)	94 (29)	93 (27)	0.459
Cardiac arrest	176 (22.2)	132 (23.1)	44 (19.9)	0.330
Sinus rythm	565 (72.3)	404 (71.6)	161 (74.2)	0.473
LVEF (%)	32 (13)	31 (13)	34 (13)	0.003
Confusion at presentation	486 (61.4)	352 (61.8)	134 (60.4)	0.717
Lactate				0.056
< 2 mmol/L	169 (23.8)	128 (24.9)	41 (21)	
2–4 mmol/L	213 (30)	162 (31.5)	51 (26.2)	
> 4 mmol/L	328 (46.2)	225 (43.7)	103 (52.8)	
Glucose > 191 mg/dL	357 (46.9)	252 (45.8)	105 (49.8)	0,329
eGFR (ml/min/1.73 m2)				0.027
< 30	146 (24.3)	90 (20.9)	56 (32.7)	
30–60	271 (45)	205 (47.6)	66 (38.6)	
> 60	185 (30.7)	136 (31.6)	49 (28.7)	
CardShock score value	4.7 (1.7)	4.7 (1.7)	4.8 (1.8)	0.501
IABP II score value	2.5 (1.8)	2.5 (1.8)	2.6 (1.8)	0.525

*BMI* Body mass index, *COPD* Chronic obstructive pulmonary disease, *PAD* Peripheral artery disease, *MI* Myocardial infarction, *PCI* percutaneous coronary intervention, *CABG* Coronary artery bypass grafting, *ACS* Acute Coronary Syndrome, *LVEF* Left Ventricle Ejection Fraction, *eGFR* estimated glomerular filtration rate

### Clinical characteristics according to gender status

**Fig. 1:**
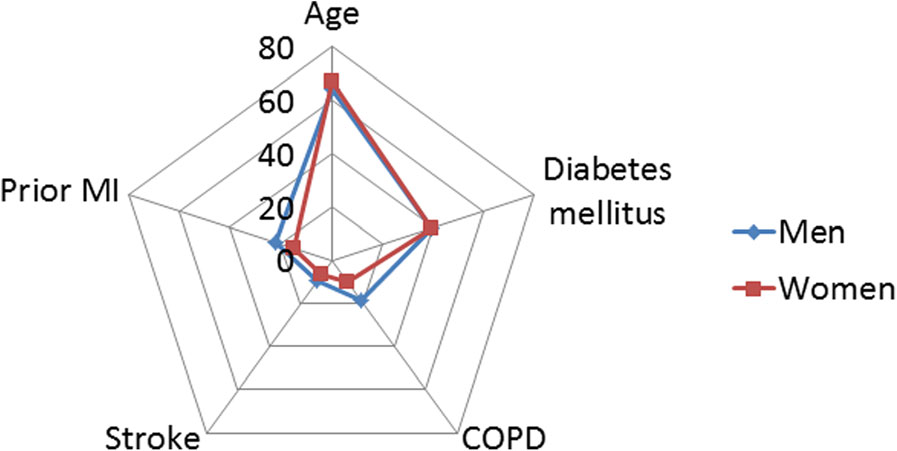
Baseline clinical characteristics according to gender status

Women were significantly older, were less often active smokers and had a significantly lower proportion of comorbidities such as chronic obstructive pulmonary disease (COPD) and prior MI (Fig. 1). CS was less often related to ACS in women. A slightly better left ventricular function was observed in this group, as well as lower values of lactate at admission and higher values of eGFR. No significant differences were observed regading the CardShock and IABP II risk scores values according to gender status.

### Clinical management according to gender status

The proportion of use of vasoactive drugs was not significantly different in both groups, except for a less common utilisation of dobutamine and a higher prescription of milrinone in women. Likewise, the rate of utilisation of in-hospital invasive procedures (renal replacement therapy, invasive ventilation, therapeutic hypothermia) was similar between both groups. Mechanical circulatory support was also used with a similar proportion in men and women (Table 2).

**Table 2:** Clinical management according to gender status

	Whole cohort (*n* = 793)	Male (*n* = 571)	Female (*n* = 222)	*P* value
Vasoactive drugs				
Dobutamine	716 (90.3)	530 (92.8)	186 (83.8)	0.001
Dopamine	62 (7.8)	42 (7.4)	20 (9)	0.435
Adrenaline	132 (16.6)	95 (16.6)	37 (16.7)	0.992
Noradrenaline	620 (78.2)	444 (77.8)	176 (79.3)	0.436
Levosimendan	75 (9.5)	59 (10.3)	16 (7.2)	0.177
Milrinone	16 (2)	6 (1.1)	10 (4.5)	0.004
Invasive procedures				
Renal replacement therapy	150 (19)	105 (18.5)	45 (20.3)	0.558
Invasive mechanical ventilation	489 (61.7)	358 (62.7)	131 (59)	0.617
Non invasive mechanical ventilation	77 (9.7)	53 (9.3)	24 (10.8)	0.597
Therapeutic hypothermia	43 (5.4)	35 (6.1)	8 (3.6)	0.158
Swan-Ganz catheter	273 (34.4)	191 (33.5)	82 (36.9)	0.353
Intraaortic counterpulsation	370 (46.7)	274 (48)	96 (43.2)	0.229
ECMO	67 (8.4)	53 (9.3)	14 (6.3)	0.176
Impella®	30 (3.8)	23 (4)	7 (3.2)	0.562
Levitronix centrimag®	55 (6.9)	40 (7)	15 (6.8)	0.902
Revascularization				0.426
None	334 (42.1)	233 (40.8)	101 (45.5)	
PCI	433 (54.6)	320 (56)	113 (50.9)	
Surgical	26 (3.3)	18 (3.2)	8 (3.6)	

*PCI* Percutaneous coronary intervention

### In-hospital mortality and predictive ability of Cardshock and IABP-II risk scores according to gender

In-hospital mortality for the overall cohort was 346/793 (43.6%). Mortality was not significantly different according to gender (47.3% vs 42.2%, *p* = 0.194). Mortality was more commonly due to cardiac causes (76.2%, vs 61.4%) and less commonly due to severe neurologic damage in men (4.8% vs 12%, *p* = 0.027).

Cardshock risk score showed a good ability for predicting in-hospital mortality both in man (AUC 0.69, 95% CI 0.6547–0.739) and women (AUC 0.735, 95% CI 0.663–0.806). Likewise, the IABP-II successfully predicted in-hospital mortality in both groups (AUC in man 0.693, 95% CI 0.647–0.739; women AUC 0.722, 95% CI 0.649–0.795). The predictive ability of both scores was not significantly different according to gender.

**Fig. 2:**
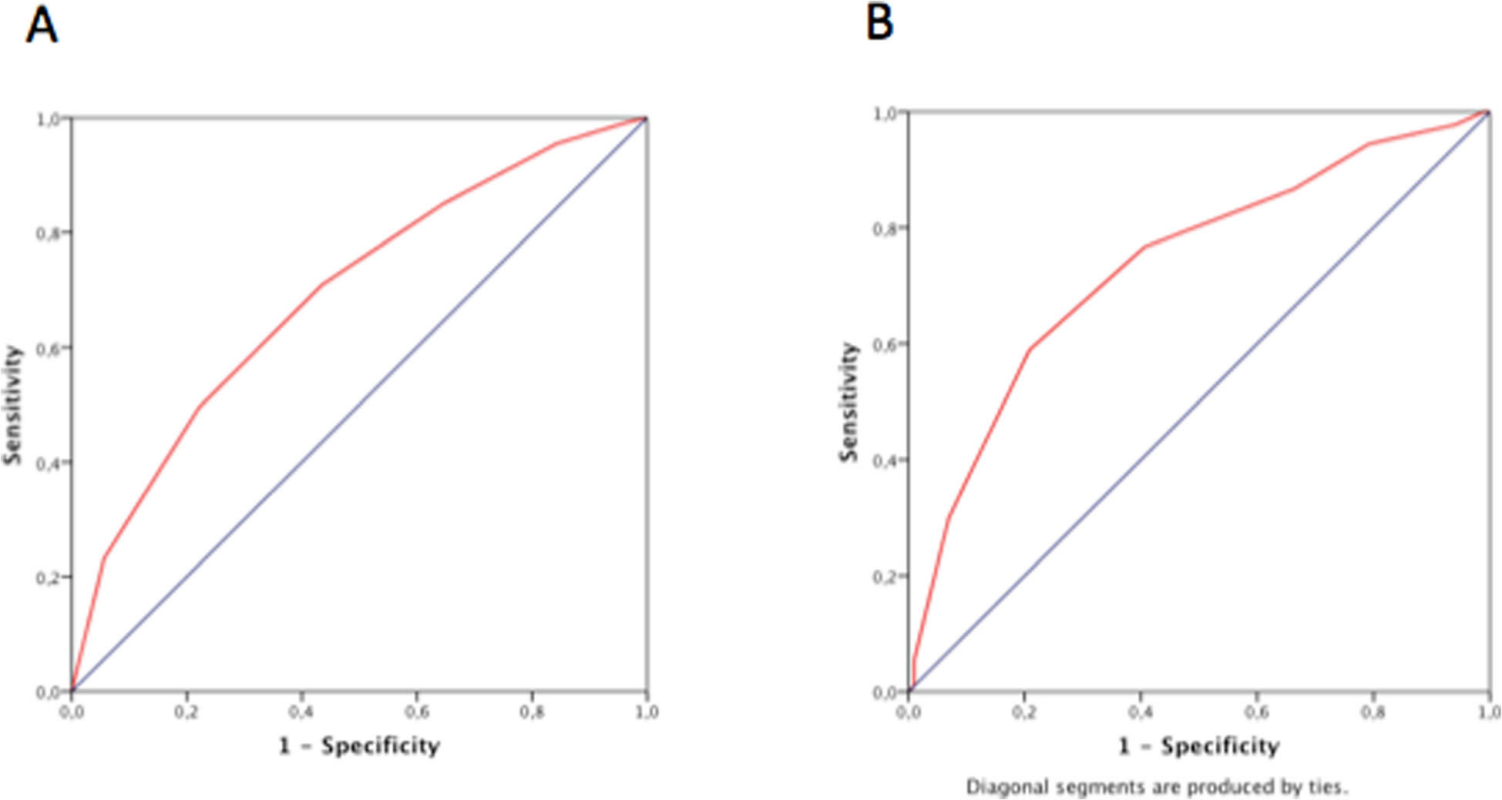
ROC curves for the prediction of in-hospital mortality of the Cardshock score in man (**a**) and women (**b**)

**Fig. 3:**
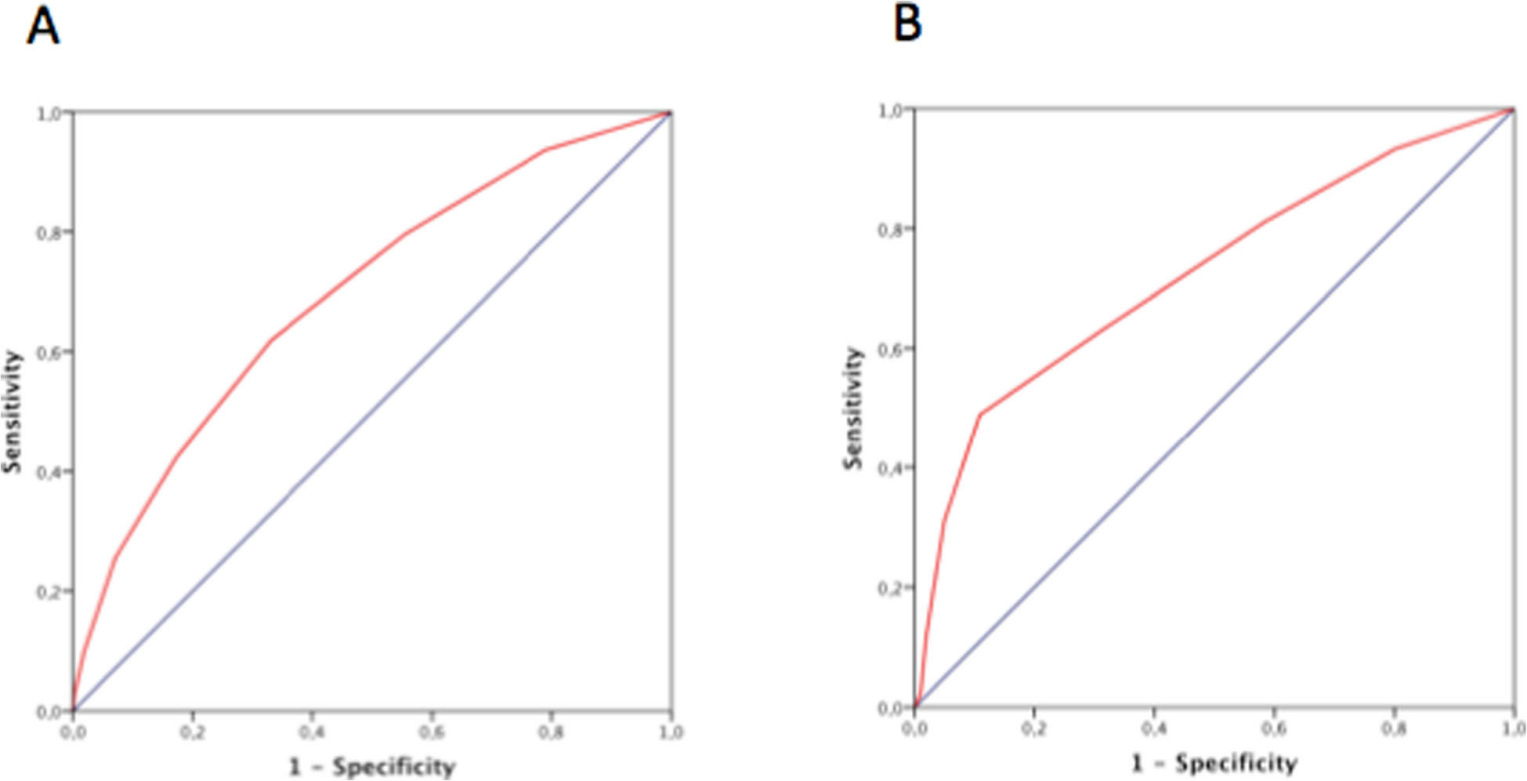
ROC curves for the prediction of in-hospital mortality of the IABP-II risk score in men (**a**) and women (**b**)

Calibration was also acceptable for both scores in both groups (supplementary Table 1). Figure 2 shows the ROC curves for the prediction of in-hospital mortality of the Cardshock score in man (2a) and women (2b). Figure 3 shows the ROC curves for the prediction of in-hospital mortality of the IABP-II risk score in men (3a) and women (3b).

### Contribution of each components of the Cardshock and IABP-II scores to in-hospital mortality according to gender

In man, the components of the Cardshock with a stronger association with mortality were confusion at presentation, lactate levels and glomerular filtration. Left ventricular disfunction was also significantly associated with mortality, while the association between age > 75 years and mortality had a non-significant trend. In contrast, the association between previous MI or coronary artery bypass surgery and ACS etiology with mortality was clearly not significant (supplementary Table 2). In contrast, in women only lactate levels and glomerular filtration were significantly associated with mortality (supplementary Table 2).

Likewise, significant differences were observed regarding the specific contribution of each of the components of the IABP-II score for predicting mortality according to gender. While in man age, creatinine, lactate levels and TIMI flow grade in culprit lesion were significantly associated to in-hospital mortality, in women this association with mortality was observed only for creatinine and lactate levels (supplementary Table 3).

## Discussion

Main findings from this study are: a) around one of each three of these patients with CS from routine clinical practice were women; b) female patients with CS were older, and CS was less commonly ACS-related in this group; c) both clinical management and in-hospital mortality were similar regardless gender status, and d) both the CardShock and IABP-II risk scores depicted a good ability for predicting mortality in both sexes.

CS is a severe clinical condition with a very high mortality despite continuous therapeutic advances. Clinical evidence regarding the optimal management in these complex patients is scarce. The few randomized trials [3, 14, 15] addressing patients with CS showed negative results, and literature is mostly base in heterogeneous observational studies addressing ACS-related CS [16–21]. The proportion of women in these series ranges between 25 and 45%, with mean ages ranging between 65 and 80 years [16–22].

This series included one of the youngest populations of CS patients, with a relatively low proportion of women as compared to previous data. This could be related to the fact that this study included highly selected patients from high complexity centers, with consolidated heart transplantation and advanced heart failure programs in most cases. Importantly, almost 40% of patients had non-ACS related CS, in contrast to most published CS series [16–22].

Women with cardiovascular disease have been clearly under represented in clinical trials, and the evidence regarding their optimal clinical management in different clinical scenarios is scarce, especially in patients with ACS. Most data come from observational studies showing that women are usually older, have a high degree of comorbidities, a lower adherence to current recommendations and a higher mortality [10, 11]. Information about differences in clinical characteristics, management and outcomes according to gender in CS patients is scarce. Most of the few series show that women with CS are usually older [1, 16–22]. However, differences regarding the proportion of comorbidities between men and women are not so evident as compared to patients with ACS. This is especially remarkable in series of patients undergoing advanced therapies at high complexity centers, probably because of a likely selection bias.

In an interesting contribution, Hayıroğlu et al. [23] assessed predictors of in-hospital mortality in 319 patients with MI complicated with CS. Despite a trend to a higher proportion of women was observed among non-survivors, gender showed no significant association with mortality in the multivariate analysis. Likewise, in a series of 544 patients with STEMI with CS undergoing primary PCI, Cheng et al. [24] described a significant association between female gender and mortality in univariable analysis. However, only age, baseline lactate creatinine levels remained independent predictors of 30-day mortality in the multivariable analysis.

Women from this series were significantly older, but the proportion of important comorbidities such as diabetes, prior stroke or peripheral artery disease was not significantly different between both groups. Indeed, the proportion of comorbidities such as COPD and prior MI was lower in women. Interestingly, the proportion of ACS-related CS was significantly lower in women.

A significant concern exists about the lower adherence to recommendations in women with cardiovascular disease, especially in ACS. Likewise, in women with acute heart failure intensive medical and interventional therapies are also underutilized [25]. Information about management disparities in women with CS is scarce. Abdel-Qadir et al. [20] assessed a series of 9750 patients with ACS-related CS from the Ontario Myocardial Infarction Database. The authors described that women with CS were less likely to present to revascularization-capable sites, were less often revascularized and less likely to be transferred when they presented to non-revascularization sites.

Improving this gender related disparities is one of the major challenges for the upcoming years. In this sense, the implementation of standardized ST segment elevation myocardial infarction (STEMI) protocol and Systems has been associated to a reduction of health care differences and mortality in women with ACS [26]. On the other hand, management at high volume specialized centers has been associated to a better adherence to recommendations and lower mortality in patients with CS [27]. In an interesting study, Iantorno et al. [21] assessed a series of 8845 consecutive adult patients admitted to a tertiary coronary care unit, of whom 42.1% were women. A similar pulmonary artery catheter (PAC) use according to gender was observed (11.3% in women and 11.5% in men). In CS patients, PAC use rates in women and men were also similar (50.3% vs 49.1%) The authors concluded that full-time intensivist staffing might contribute to reduce gender-based treatment disparities.

As stated before, management disparities seem to be lower in highly selected patients requiring advanced support measures. Joseph et al. [18] described a series of 180 patients from the cVAD Registry who underwent percutaneous coronary intervention (PCI) and Impella 2.5 support for CS complicating an acute MI, of whom 49 (27.2%) were women. Despite women were older, the proportion of important comorbidities such as diabetes, prior MI, PAD or renal dysfunction was not significantly different according to gender. No differences in timing to intervention were found between men and women. The number of inotropes and the proportion of mechanical ventilation and intraaortic counterpulsation were not significantly different in both groups. There was no difference in survival to discharge (*p* = 0.3). Interestingly, the magnitude of the survival benefit was significantly greater in women who received the Impella pre-PCI as compared to men.

Consistently with these data, we did not observe in our patients significant differences regarding vasoactive drugs prescription and use of in-hospital invasive procedures according to gender. In our opinion, this might be mostly related to the high degree of selection of CS patients from this series (especially women with CS), as well as the fact of being treated at high complexity academic hospitals. This similar management patterns according to gender might have contributed to the similar mortality observed in both groups.

Patients with stablished shock criteria and high lactate levels have a significantly higher mortality. Therefore, early risk stratification is crucial step in order to select the optimal clinical management. Recently two risk scores have been published to predict short-term mortality in patients with CS: the CardShock risk score [1], derived from a multicentre cohort of ACS and non-ACS patients, and the IABP-SHOCK II risk score [5], derived from a clinical trial performed in acute MI patients undergoing percutaneous coronary intervention (PCI). Both scores have shown good discrimination for short-term mortality. It is important to note that gender is not a component in either CardShock or IABP-SHOCK risk scores. To our knowledge no study addressed the potential disparities of risk stratification according to gender in patients with CS. This is an interesting point, since women were under represented in the populations form whom both scores were derived. Data from this study showed a good ability for predicting in-hospital mortality from both scores in both women and men. Therefore, both the Cardshock and the IABP-II scores should be considered useful tools also in women with CS.

Other predictive factors have been described in patients with CS. Information for the coronary anatomy can also contribute to predict outcomes in CS patients. In this sense, recently a combination of clinical and angiographic factors by the SYNTAX score II showed also a significant predictive ability for predicting mortality in STEMI patients with CS undergoing primary PCI [28].

This study has some limitations, such as its moderate sample size and its observational nature, so we cannot rule the effect of unmeasured confounding. Patients were mostly admitted to high complexity hospitals with heart transplantation and advanced heart failure programs, which may lead to a significant selection bias. Therefore, our findings should be validated in larger series of non-selected CS patients admitted to other type of centers. However, we believe that despite these limitations this study retrieves novel and interesting data about the clinical picture, management and risk stratification of patients with CS according to gender. Improving clinical outcomes of these complex patients may lead to potential important social and economic consequences.

## Conclusions

About one of each three of these patients with CS from routine clinical practice were women. Female patients with CS were older, and CS was less commonly ACS-related in this group. No significant differences were observed regarding management and in-hospital mortality according to gender. Both the CardShock and IABP-II risk scores depicted a good ability for predicting mortality also in women with CS.

## Supplementary information

**Additional file 1: Table S1.** Predictive contribution to in-hospital mortality of each of the components of the CardShock score according to gender status **Table S2.** Predictive contribution to in-hospital mortality of each of the components of the IABP-II score according to gender status **Table S3.** Discrimination and calibration of the CardShock and IABP II scores for predicting in-hospital mortality according to gender status.

## Data Availability

The datasets used and/or analyzed during the current study are available from the corresponding author on reasonable request.

## Abbreviations

CS: Cardiogenic shock
MI: Myocardial infarction
ACS: Acute coronary syndromes
TIMI flow: Thrombolysis in myocardial infarction
ROC curves: Receiver operating characteristic
AUC: Area under the curve
eGFR: Estimated glomerular filtration
COPD: Chronic obstructive pulmonary disease
STEMI: ST segment elevation myocardial infarction
PAC: Pulmonary artery catheter
PCI: Percutaneous coronary intervention
