# Supplementary material for: Clinical picture, management and risk stratification in patients with cardiogenic shock: does gender matter?
Source: BMC Cardiovasc Disord. 2020 Apr 21;20:189. doi: 10.1186/s12872-020-01467-4 (PMC7362401; doi:10.1186/s12872-020-01467-4)
Supplement: Supplementary file 1 — Additional file 1: Table S1. Predictive contribution to in-hospital mortality of each of the components of the CardShock score according to gender status Table S2. Predictive contribution to in-hospital mortality of each of the components of the IABP-II score according to gender status Table S3. Discrimination and calibration of the CardShock and IABP II scores for predicting in-hospital mortality according to gender status. [file 12872_2020_1467_MOESM1_ESM.docx]

Supplementary table 1. Predictive contribution to in-hospital mortality of each of the components of the CardShock score according to gender status

|  | Male | | Female | |
| --- | --- | --- | --- | --- |
|  | OR (95% CI) | p value | OR (95% CI) | p value |
| Age >75 years | 1.51 (0.97-2.35) | 0.067 | 1.45 (0.73-2.84) | 0.287 |
| Confusion at presentation | 1.91 (1.28-2.84) | 0.001 | 1.33 (0.67-2.62) | 0.416 |
| Previous MI or CABG | 0.94 (0.59-1.49) | 0.791 | 1.02 (0.41-2.53) | 0.973 |
| ACS aetiology | 1.16 (0.77-1.74) | 0.483 | 1.11 (0.57-2.18) | 0.757 |
| LVEF <40% | 1.59(1.01-2.50) | 0.046 | 1.07 (0.55-2.09) | 0.849 |
| Lactate  2-4 mmol/L  >4 mmol/L | 1.55 (0.91-2.65)  2.93 (1.78-4.81) | 0.001 | 1.64 (0.61-4.43)  4.98 (1.99-12.4) | 0.001 |
| eGFR  30-60 ml/min/1.73m2  <30 ml/min/1.73m2 | 1.66 (1.03-2.65)  2.90 (1.68-4.99) | 0.001 | 1.33 (0.55-3.24)  3.35 (1.34-8.38) | 0.012 |

MI: Myocardial infarction; CABG: Coronary artery bypass grafting; ACS: Acute Coronary Syndrome; LVEF: Left Ventricle Ejection Fraction; eGFR: estimated glomerular filtration rate.

Supplementary table 2. Predictive contribution to in-hospital mortality of each of the components of the IABP-II score according to gender status

|  | Male | | Female | |
| --- | --- | --- | --- | --- |
|  | OR (95% CI) | p value | OR (95% CI) | p value |
| Age >73 years | 2.02 (1.33-3.07) | 0.001 | 1.57 (0.82-3.01) | 0.176 |
| History of stroke | 0.78 (0.55-1-10) | 0.159 | 1.15 (0.61-2.20) | 0.658 |
| Glucose >10.6 mmol/L | 1.29 (0.87-1.92) | 0.197 | 1.72 (0.88-3.37) | 0.116 |
| Creatinine >132.6 | 2.56 (1.72-3.79) | 0.001 | 2.48 (1.27-4.86) | 0.008 |
| Lactate > 5 mmol/L | 1.67 (1.36-2.04) | 0.001 | 1.98 (1.42-2.76) | 0.001 |
| TIMI flow grade <3 after PCI | 1.45 (1.10-1.91) | 0.008 | 1.19 (0.78-1.84) | 0.418 |

Supplementary table 3. Discrimination and calibration of the CardShock and IABP II scores for predicting in-hospital mortality according to gender status

|  | Male | | | Female | | |
| --- | --- | --- | --- | --- | --- | --- |
|  | AUC (95% CI) | p value | HL test (p) | AUC (95% CI) | p value | HL test (p) |
| Cardshock score | 0.691 (0.645-0.737) | 0.001 | 0.695 | 0.735 (0.663-0.806) | 0.001 | 0.419 |
| IABP II score | 0.693 (0.647-0.739) | 0.001 | 0.971 | 0.722 (0.649-0.795) | 0.001 | 0.401 |
